# Screening of FDA-Approved Drugs for Inhibitors of Japanese Encephalitis Virus Infection

**DOI:** 10.1128/JVI.01055-17

**Published:** 2017-10-13

**Authors:** Shaobo Wang, Yang Liu, Jiao Guo, Peilin Wang, Leike Zhang, Gengfu Xiao, Wei Wang

**Affiliations:** a State Key Laboratory of Virology, Wuhan Institute of Virology, Chinese Academy of Sciences, Wuhan, China; b University of the Chinese Academy of Sciences, Beijing, China; Washington University School of Medicine

**Keywords:** FDA-approved drugs, Japanese encephalitis virus, NS4B, flavivirus, high-throughput screening, manidipine

## Abstract

Japanese encephalitis virus (JEV), an arthropod-borne flavivirus, is a major cause of acute viral encephalitis in humans. No approved drug is available for the specific treatment of JEV infections, and the available vaccines are not effective against all clinical JEV isolates. In the study described here, a high-throughput screening of an FDA-approved drug library for inhibitors of JEV was performed. Five hit drugs that inhibited JEV infection with a selective index of >10 were identified. The antiviral activities of these five hit drugs against other flavivirus, including Zika virus, were also validated. As three of the five hit drugs were calcium inhibitors, additional types of calcium inhibitors that confirmed that calcium is essential for JEV infection, most likely during viral replication, were utilized. Adaptive mutant analysis uncovered that replacement of Q130, located in transmembrane domain 3 of the nonstructural NS4B protein, which is relatively conserved in flaviviruses, with R or K conferred JEV resistance to manidipine, a voltage-gated Ca^2+^ channel (VGCC) inhibitor, without an apparent loss of the viral growth profile. Furthermore, manidipine was indicated to protect mice against JEV-induced lethality by decreasing the viral load in the brain, while it abrogated the histopathological changes associated with JEV infection. This study provides five antiflavivirus candidates and identifies cytoplasmic calcium to be a novel antiviral target for the treatment of JEV infection. The findings reported here provide therapeutic possibilities for combating infections caused by flaviviruses.

**IMPORTANCE** No approved therapy for the treatment of Japanese encephalitis virus infection is currently available. Repurposing of approved drugs would accelerate the development of a therapeutic stratagem. In this study, we screened a library of FDA-approved drugs and identified five hit drugs, especially calcium inhibitors, exerting antiflavivirus activity that blocked viral replication. The *in vivo* efficacy and toxicity of manidipine were investigated with a mouse model of JEV infection, and the viral target was identified by generating an adaptive mutant.

## INTRODUCTION

Flaviviruses are taxonomically classified in the genus Flavivirus and family Flaviviridae. These viruses comprise over 70 different pathogens, such as Japanese encephalitis virus (JEV), Zika virus (ZIKV), dengue virus (DENV), West Nile virus (WNV), and yellow fever virus (YFV). Most flaviviruses are arthropod borne and cause public health problems worldwide (1). The development and usage of vaccines against some flaviviruses, such as JEV, YFV, and tick-borne encephalitis virus (TBEV), have decreased the rates of morbidity and mortality from infections caused by these viruses (2); however, flavivirus-induced diseases are still pandemic, and few therapies beyond intensive supportive care are currently available.

Flaviviruses have an approximately 11-kb positive-stranded RNA genome containing a single open reading frame (ORF) flanked by untranslated regions (UTRs) at both termini. The ORF encodes three structural proteins, including the capsid (C), membrane (premembrane [prM] and membrane [M]), and envelope (E), and seven nonstructural proteins (NS1, NS2A, NS2B, NS3, NS4A, NS4B, and NS5) (3). These seven nonstructural proteins participate in viral replication, virion assembly, and virus escape from immune surveillance.

To date, no specific antivirals with activity against flaviviruses are available. To address this, we conducted a screen of a library of 1,018 FDA-approved drugs. Since flaviviruses are similar in structure and pathogenesis, we first utilized JEV as the prototype to screen the drug library and subsequently validated the antiviral activities with ZIKV, WNV, and DENV type 2 (DENV-2). The hit drugs identified in this study offer potential new therapies for the treatment of flavivirus infection and disease.

## RESULTS

### Screening of an FDA-approved drug library for inhibitors of JEV infection.

Recombinant viral particles (RVPs) with the luciferase-reporting replicon enveloped by the JEV structural proteins were used to select inhibitors, with a focus on those that inhibit virus entry and replication, by a high-throughput screening (HTS) assay (4, 5). The number of genomic RNA copies of RVP was determined to be 8.4 × 10^6^ copies/ml by using a standard curve generated with plasmids carrying the infectious clone. The HTS assay conditions, including the seeding cell density and RVP dose, were optimized to be 10,000 cells per 96-well plate and 20 μl (16 copies/cell) RVP for the infective dose, respectively. Under the optimized conditions, the signal-to-basal (S/B) ratio, coefficient of variation (CV), and *Z*′ factor were 38,374, 2.8%, and 0.89, respectively, which demonstrated that the assay was robust and suitable for the large-scale screening of compounds.

**FIG 1:**
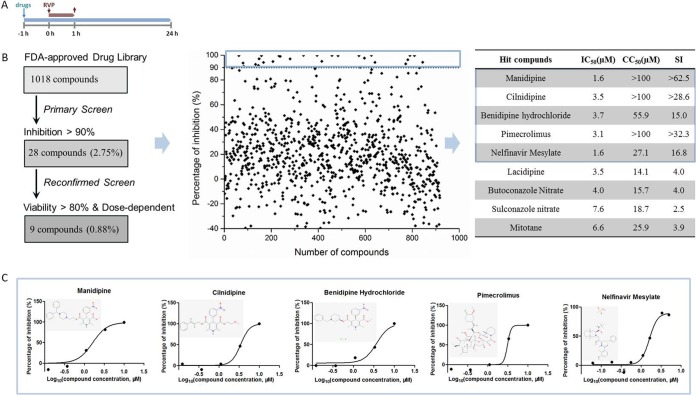
HTS for inhibitors of JEV infection from an FDA-approved drug library. (A) HTS assay timeline. Vero cells were seeded at a density of 1 × 10^4^ cells per well in 96-well plates. After overnight incubation, cells were treated in duplicate with 10 μM compounds. One hour later, 20 μl RVPs was added. The supernatant was removed 1 h later and the compounds were readded to the cells for an additional 23 h. (B) (Left) HTS assay flowchart. The criterion required for the compounds to pass the primary screen was inhibition of >90%, and 28 primary candidates were selected. In the reconfirmation screen, 9 compounds with dose-dependent inhibition and cell viability of >80% were selected. (Middle) HTS of a library of 1,018 FDA-approved drugs for primary candidates inhibiting JEV infection. Each dot represents the percent inhibition achieved with each compound at a concentration of 10 μM. The dots located in the blue box represent inhibition of >90%. (Right) IC_50_s, CC_50_s, and SIs of nine compounds selected from the reconfirmation screen. The top five compounds depicted in the blue box showed SIs of greater than 10 and were designated hit drugs. (C) Dose-response curves of the five hit drugs for inhibition of JEV RVP infection. (Insets) The structure of each drug.

A schematic of the HTS assay is depicted in Fig. 1B. After three rounds of screening, five hits with a selective index (SI; which is equal to the 50% cytotoxic concentration [CC_50_[/50% inhibitory concentration [IC_50_]) of >10 were selected. The CC_50_ values of the hit drugs exhibited in Fig. 1B were similar to those previously published for diverse cell systems but determined using different toxicity assays (6–13). Three of the hit drugs, manidipine, cilnidipine, and benidipine hydrochloride, were dihydropyridine (DHP) voltage-gated Ca^2+^ channel (VGCC) antagonists, while pimecrolimus is an inhibitor of inflammatory cytokine secretion and nelfinavir mesylate is an HIV-1 protease blocker. All five drugs exhibited a dose-dependent inhibition of JEV RVP infection (Fig. 1C). To validate the antiviral effect, hit drugs were purchased from other commercial sources and tested. In the reconfirmation screen, all hit drugs showed antiviral and cytotoxic effects similar to those found in the primary screen.

### Validation of hit drugs.

**FIG 2:**
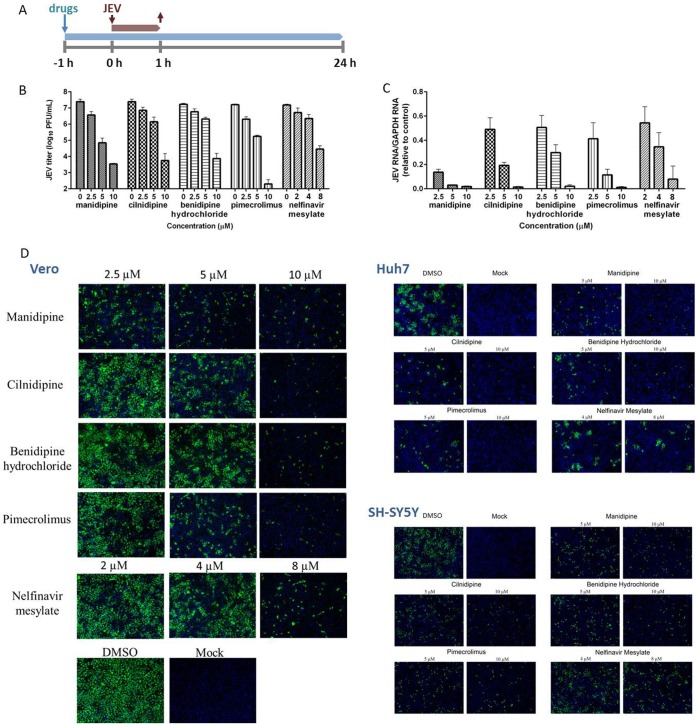
Validation of the antiviral effects of the hit drugs. (A) Antiviral assay timeline. Vero cells were incubated with the hit drugs from 1 h preinfection and then infected with JEV at a multiplicity of infection of 0.2 for 1 h. (B to D) Twenty-four hours later, the cell culture supernatants were subjected to the viral titer assay (B), while cell lysates were assessed for quantification of the amount of viral RNA (C), and duplicate cells were analyzed for protein expression by IFA (D). IFA images showing the viral prM protein (green) and nuclei (blue) are displayed for Vero, Huh-7, and SH-SY5Y cells. Data are represented as the means ± SDs from 3 independent experiments. GAPDH, glyceraldehyde-3-phosphate dehydrogenase.

To verify the results obtained by the luciferase reporter assays, we also investigated the antiviral effect of the five hit drugs on wild-type JEV strain AT31. As expected from the HTS assay, all five drugs robustly inhibited virus production, with a reduction of approximately 4 to 5 log units at the highest concentration and an approximately 1-log-unit decrease with 2.5 μM the drugs (Fig. 2B). A sharp decrease in JEV RNA levels was also detected (Fig. 2C). The attenuated RNA levels in the high-dose, middle-dose, and low-dose groups were all above 40%. In particular, in the manidipine-treated group, the inhibitory effect was at least 80% compared to that for the control, which showed a strong inhibition of viral replication. Consistent with the inhibition of virus replication and production, expression of the viral structural protein prM was hardly detectable following treatment with the drugs at the high concentration (Fig. 2D). Overall, the results in Fig. 2 confirmed that the five hit drugs inhibited JEV infection in a dose-dependent manner *in vitro*.

### Drugs inhibit JEV infection during viral RNA synthesis.

**FIG 3:**
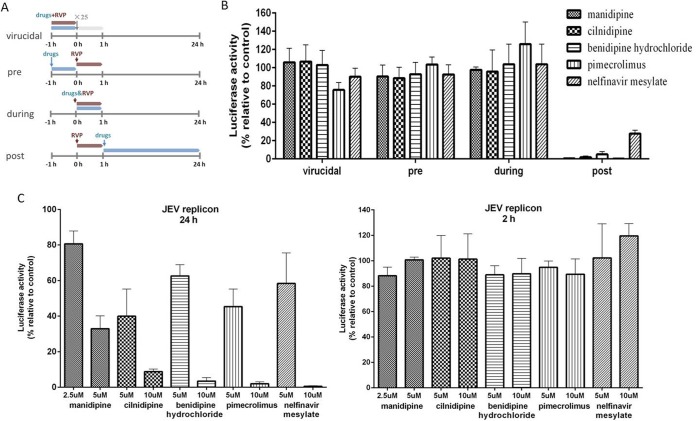
Time-of-addition analysis of the antiviral activity of the hit drugs. (A) Schematic illustration of time-of-addition experiment. (B) Vero cells were infected with JEV RVP for 1 h (0 to 1 h). Manidipine (10 μM), cilnidipine (10 μM), benidipine hydrochloride (10 μM), pimecrolimus (10 μM), or nelfinavir mesylate (8 μM) was introduced at different time points of RVP infection, designated virucidal, pretreatment (pre), during treatment (during), or posttreatment (post). The inhibitory effect of the drugs in each group was determined by a luciferase activity assay. (C) BHK-21 cells transfected with the JEV replicon were treated with drugs at the indicated concentrations, and luciferase activities were determined at 24 h (left) and 2 h (right).

Because RVPs, which have a natural virus-like envelope on the outside and a replicon on the inside, permitted the quantification of JEV productive entry and replication, a time-of-addition experiment was performed to investigate whether the hit drugs blocked the entry step or the replication step. As shown in Fig. 3B, no suppression of luciferase activity by any of the hit drugs was observed when they were used as treatments before infection or during infection or as a virucide, suggesting that these drugs do not inhibit JEV infection either by inactivating the virus directly or by blocking JEV entry. However, these drugs exerted fully inhibitory effects when they were added at 1 h postinfection, suggesting that viral replication was the stage at which these drugs showed inhibitory activity.

To confirm this suggestion, we investigated the inhibitory effects of these drugs on the JEV replicon. The highest concentration of manidipine and nelfinavir mesylate tested in baby hamster kidney (BHK-21) cells was adjusted to 5 μM and 10 μM, respectively. It was shown that all five drugs inhibited JEV RNA synthesis in a dose-dependent manner, while neither drug inhibited the initial translation of replicon RNA (5, 14) (Fig. 3C), confirming that these drugs inhibited JEV infection at the stage of replication.

### Hit drugs exhibit broad-spectrum antiflavivirus activity.

**FIG 4:**
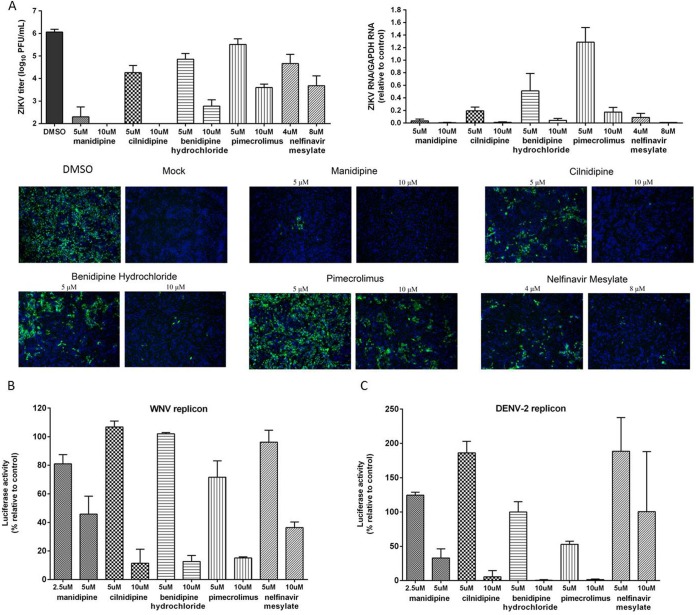
Broad-spectrum antiviral activity of the hit drugs against flaviviruses. (A) Vero cells were infected with ZIKV at a multiplicity of infection of 0.2 for 1 h. The hit drugs were added to the cells at 1 h preinfection and were present throughout the experiment. At 48 h postinfection, the cell culture supernatants were subjected to a viral titer assay (top left), while cell lysates were assessed for viral RNA quantification (top right), and duplicate cells were analyzed by IFA for E protein (green) expression (bottom). Nuclei (blue) were stained with DAPI (4′,6-diamidino-2-phenylindole). (B and C) BHK-21 cells transfected with the WNV or DENV-2 replicon were treated with the hit drugs at the indicated concentrations, and luciferase activities were determined 48 h or 72 h later.

In order to determine whether the antiviral activity of the five hit drugs extended to other flaviviruses, we explored their antiviral effect against ZIKV. Similar to the findings for JEV, the ZIKV titer was decreased by multiple log units when ZIKV was treated with a high concentration of each of the drugs (Fig. 4A). Moreover, ZIKV exhibited a higher sensitivity to the two calcium channels inhibitors manidipine and cilnidipine than JEV, with no plaque formation being observed at 10 μM. Consistent with this result, sharp decreases in the level of replication of ZIKV RNA and the level of expression of viral protein were also detected (Fig. 4A). Notably, treatment with 5 μM manidipine produced a 95% inhibition of viral replication, translation, and viral yields. Taken together, these results indicate that the hit drugs could effectively inhibit ZIKV infection.

Since these drugs exhibited their anti-JEV effects at the stage of viral replication, we further tested the effects against WNV and DENV-2 by using WNV and DENV-2 replicons. Similar to the results for JEV, a dose-dependent reduction in the level of WNV replication was observed with the drug treatments. The same phenotype was observed for DENV-2 for all drugs except nelfinavir mesylate, which showed no effect at the concentrations tested (Fig. 4B and C). Together, these results indicate that the five hit drugs are excellent candidates for broad-spectrum antiflavivirus treatment.

### Antiviral effect of calcium inhibitors.

**FIG 5:**
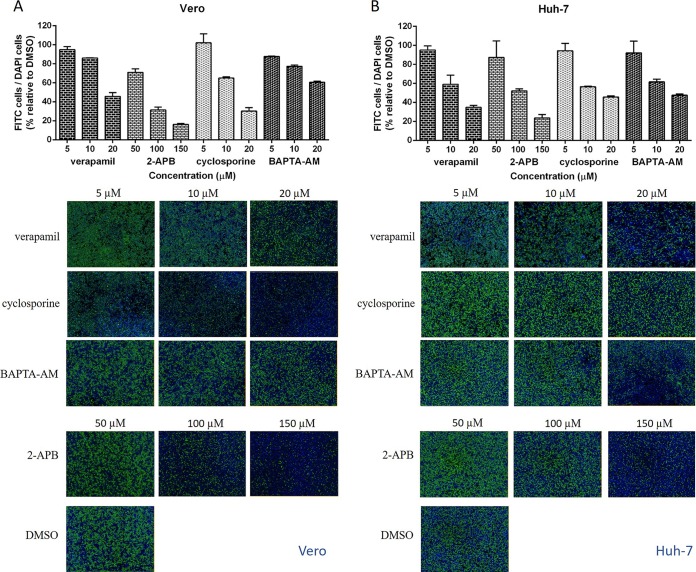
The inhibitory effects of four kinds of calcium inhibitors, verapamil, 2-APB, cyclosporine, and BAPTA-AM, on JEV infection on Vero (A) and Huh-7 (B) cells. (Top) The ratio of the total number of cells stained with fluorescein isothiocyanate (FITC; green; JEV prM) to the number stained with DAPI (blue; nuclei) was calculated by comparison to that for the DMSO-treated control group. (Bottom) The IFA was monitored by use of an Operetta high-content imaging system (PerkinElmer). The experiment was performed as described in the legend to Fig. 2A. Data are represented as the means ± SDs from 4 independent experiments.

Since three hit drugs, manidipine, cilnidipine, and benidipine hydrochloride, were DHP VGCC inhibitors, we asked whether other calcium antagonists could block JEV infection. To address this question, we employed four different classes of inhibitors. Verapamil, a prototype phenylalkylamine (PAA) VGCC inhibitor (15), exhibited a dose-dependent inhibition of JEV on both African Green monkey kidney (Vero) and human hepatocellular carcinoma (Huh-7) cells (Fig. 5), which was consistent with the inhibitory effects of the DHP inhibitors, suggesting that calcium channels play an important role in JEV infection. Cyclosporine and 2-aminobiphenyl borate (2-APB), which inhibit the efflux of Ca^2+^ from the mitochondrial and endoplasmic reticulum (ER) pool, respectively (16–19), were also found to block JEV infection effectively. Similarly, treatment with the cell-permeant Ca^2+^ chelator 1,2-bis-(*o*-aminophenoxy)-ethane-*N*,*N*,*N*′,*N*′-tetraacetic acid, tetraacetoxymethyl ester (BAPTA-AM), could also suppress JEV infection. Taken together, we concluded that intracellular Ca^2+^ is essential for JEV infection and cytoplasmic calcium is a potent target for antiflavivirus treatment.

### Selection and characterization of manidipine-resistant JEV.

**FIG 6:**
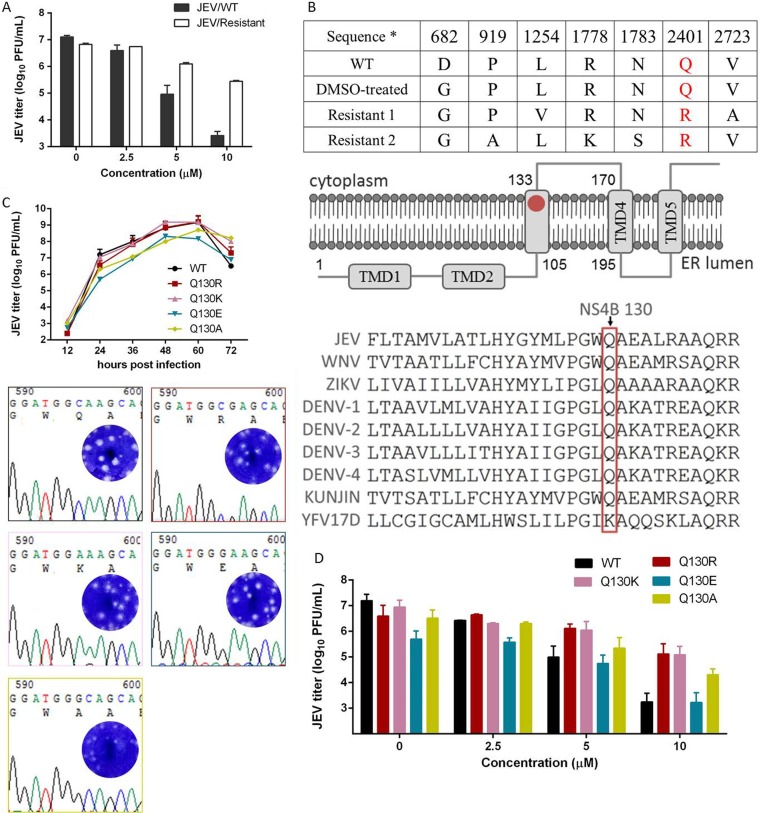
Selection and characterization of manidipine-resistant JEV. (A) The effects of manidipine on WT and P20 resistant viruses were determined in a viral titer assay. Data are represented as the means ± SDs from 3 independent assays. (B) (Top) Sequencing analysis of the mutations presented in the two resistant isolates and DMSO-treated viruses. (Middle) Membrane topology of JEV NS4B (48), with the location of Q130 highlighted with a red dot. (Bottom) Amino acid sequence alignment of the flavivirus NS4B. Q130 in NS4B is highlighted with a red box. The sequences of JEV, WNV, ZIKV, DENV-1, DENV-2, DENV-3, DENV-4, KUNJIN, and YFV 17D were derived from sequences with GenBank accession numbers BAD81042.1, AF404756, AMR39836.1, ALI16952.1, AHB63928.1, YP_001531175.2, NP_740324.1, AAP78942.1, and NP_776008.1, respectively. (C) (Top) Growth kinetics of recombinant viruses with different Q130 mutations. Vero cells were infected at a multiplicity of infection of 0.1 for 1 h. The supernatants were collected at the indicated time points postinfection and assayed for the viral titer. Data are represented as the means ± SDs for 2 independent wells. (Bottom) Sequencing chromatogram of the WT and recombinant viruses. (Insets) Plaque morphology of each virus. (D) Resistance of the recombinant viruses to manidipine. The experiment was performed as described in the legend to Fig. 2A. Data are represented as the means ± SDs from 2 independent experiments.

To identify the viral target of the calcium channel inhibitor, we selected a manidipine-resistant virus by serially passaging JEV in the presence of manidipine. Viruses from passage 20 (P20) showed robust resistance compared with the wild type (WT) (Fig. 6A). When JEV from P20 was treated with 5 μM or 10 μM manidipine, the viral titer was about 10- and 100-fold higher than that of the WT, respectively.

Individual virus clones were isolated, and two isolates were randomly selected and amplified. An amino acid substitution was observed in two isolated clones, resulting in a glutamine (Q)-to-arginine (R) switch at amino acid position 130 in transmembrane domain 3 (TMD3) of NS4B, i.e., position 2401 of the translated polyprotein in the JEV infectious cDNA clone (Fig. 6B). Sequence alignment of NS4B indicated that Q130 was conserved in all flaviviruses except YFV, which possessed a lysine at that position (Fig. 6B). The conserved Q130 of NS4B may account for the sensitivity of JEV, ZIKV, WNV, and DENV-2 to manidipine, as described above (Fig. 4), while YFV showed resistance to the drug (data not shown).

To confirm that the Q130R mutation did confer manidipine resistance and to investigate the role of Q130 in NS4B function, we produced JEV clones with the Q130R, Q130K, Q130E, or Q130A mutation by introducing the desired mutations into the infectious cDNA clone and rescuing the mutant viruses. To investigate the biological properties of the mutant viruses, we first examined the growth kinetics of the rescued viruses. As shown in Fig. 6C, all mutant viruses had an accumulation of infectious virions and reached the highest titer at 60 h postinfection. Infection of the Q130R and Q130K mutant viruses resulted in growth curves similar to the growth curve for the WT (Fig. 6C), while the Q130E and Q130A mutants produced smaller amounts of viruses between 24 and 60 h. Analysis of the plaque morphology revealed that the plaques of the Q130R, Q130K, and Q130E mutants were similar to the plaques of the WT, whereas the plaques of the Q130A mutant were smaller than those of the WT.

We next investigated the sensitivity of the four mutant viruses to manidipine. As shown in Fig. 6D, the Q130R and Q130K mutant viruses were resistant to manidipine. At a 10 μM concentration, manidipine efficiently inhibited WT JEV infection and reduced the viral yields by approximately 4 log units, while the Q130R and Q130K mutant viruses were resistant to manidipine and the viral titer decreased less than 2 log units. The Q130A mutant virus demonstrated moderate resistance and a slightly higher than 2-log-unit decrease in titer, while the Q130E mutant virus showed no resistance to manidipine.

Taken together, it could be concluded that Q130 not only is critical for conferring manidipine sensitivity but also is important for JEV replication. The replacement of glutamine with basic amino acids conferred resistance to manidipine without an apparent loss of growth.

### *In vivo* efficacy of manidipine.

**FIG 7:**
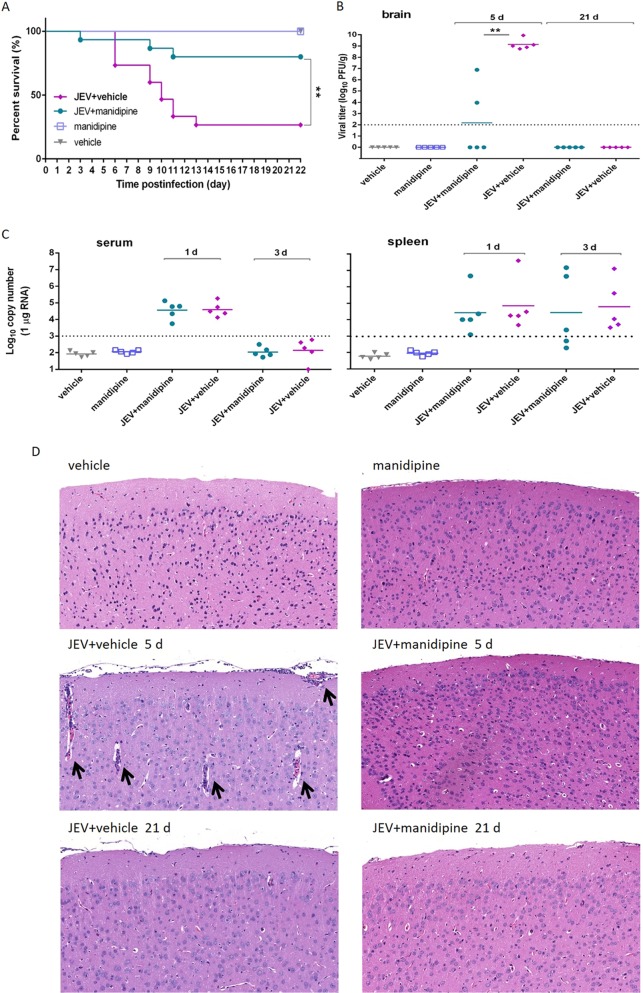
Manidipine protected mice from JEV infection. (A) Survival of mice in each group monitored for 21 days after inoculation of JEV by intraperitoneal injection. Data are shown as Kaplan-Meier survival curves (*n* = 15 for each group). (B) The viral loads in mouse brains were measured by plaque assay on days 5 and 21, respectively. (C) The viral loads in serum and spleen were measured by qRT-PCR on days 1 and 3, respectively. (D) Manidipine treatment alleviated the histopathological changes in mice caused by JEV infection. Arrows, histopathological changes, such as meningitis, perivascular cuffing, and glial nodules; dashed lines, limit of detection; d, day. **, *P* < 0.01.

As manidipine exhibited the strongest inhibitory activities on JEV replication as well as ZIKV infection when its activities were compared with those of the five hit drugs (Fig. 2 and 4A), we further examined the protective effect of manidipine against JEV-induced lethality in a mouse model. As anticipated, mice in the JEV-infected vehicle-treated group started to show symptoms, including limb paralysis, restriction of movement, piloerection, body stiffening, and whole-body tremor, from day 5 postinfection. Within 21 days postinfection, most mice in the JEV-infected group succumbed to the infection, with the mortality rate being 73% (4 out of 15 animals survived). Manidipine treatment following JEV infection reduced the mortality rate to 20% (12 out of 15 animals survived) (Fig. 7A). Mice treated with manidipine alone or treated with manidipine and infected with JEV showed little abnormal behavior, similar to the findings for the mice in the vehicle-treated group. These results suggest that manidipine provided effective protection against JEV-induced mortality.

To further relate these protective effects to the viral load and histopathological changes in the mouse brains, the viral titer was determined and mouse brain sections were collected and assayed at day 5 and day 21 postinfection, since mice started to show symptoms of JEV infection from day 5 postinfection and most of the surviving mice had recovered at day 21. The results indicated that, during the progression of the disease, manidipine treatment significantly reduced the viral load in infected mice compared to that in infected mice not receiving treatment, while no plaques formed in either the manidipine- or vehicle-treated group, and viral loads were undetectable in each group on day 21 postinfection (Fig. 7B). As JEV was rapidly cleared from the blood after inoculation and was present in the lymphatic system during the preclinical phase, the effects of manidipine on infection of serum and the spleen were evaluated at earlier time points to detect whether the drug reduced the peripheral viral loads (20, 21). As shown in Fig. 7C, manidipine had little effect on peripheral JEV infection, which indicated that manidipine protected the mice against JEV-induced lethality by decreasing the viral load in the brain. Similarly, apparent damage in the brain, including meningitis, perivascular cuffing, vacuolar degeneration, and glial nodules, was observed in the JEV-infected and vehicle-treated group on day 5 postinfection, while manidipine treatment remarkably alleviated these phenomena (Fig. 7D). These results indicate that the alleviation of histopathological changes was accompanied by a reduction in the viral load as well as a reduction in the rate of mortality, further confirming the curative effects of manidipine on viral encephalitis.

## DISCUSSION

Among the five hit drugs, manidipine, cilnidipine, and benidipine hydrochloride were VGCC inhibitors. It has been well documented in the literature that Ca^2+^ inhibitors serve to inhibit virus infection at the stage of either entry (15, 22) or replication (18) and even at the stage of budding (23). To this end, we first reviewed all 21 calcium inhibitors included in the current library of FDA-approved drugs and found that, in addition to the four DHP VGCC inhibitors listed in Fig. 1B, two other calcium inhibitors, i.e., flunarizine dihydrochloride and lomerizine hydrochloride, were also identified to be primary candidates with levels of inhibition of >90%. Similarly, three calcium channel antagonists, nisoldipine, felodipine, and nicardipine hydrochloride, showed levels of inhibition of 75%, 72%, and 66%, respectively, in the primary screen. Together, 9 of the 21 calcium inhibitors in the library, accounting for nearly half of the calcium inhibitors, exhibited levels of flavivirus inhibition of greater than 50%, suggesting that calcium, especially the calcium channel, is a potential antiviral target. To address this, another type of VGCC inhibitor, verapamil, an FDA-approved drug not yet included in the drug library used in this study, was investigated. Likewise, a Ca^2+^ chelator, BAPTA-AM, as well as the Ca^2+^ inhibitors 2-APB and cyclosporine, targeting ER and the mitochondrial Ca^2+^ channel, respectively, were employed to investigate the response of JEV infection to the decrease in intracellular Ca^2+^ levels. In line with the activities of the three hit DHP VGCC inhibitor drugs, the additional Ca^2+^ inhibitors exerted anti-JEV activity, which indicated that Ca^2+^ is indispensable for JEV infection. Thus, Ca^2+^ inhibitors might be utilized as effective treatments for flavivirus infection.

As the hit drugs exerted full inhibitory activity when they were added posttreatment, we believe that Ca^2+^ is important for flavivirus genome replication. Furthermore, selection and genetic analysis of drug-resistant viruses revealed that NS4B is the viral target of manidipine. NS4B is part of the viral replication complex and is supposed to anchor the viral replicase to the ER membrane (24). Meanwhile, the N-terminal 125-amino-acid domain of DENV NS4B was indicated to be responsible for inhibition of the immune response (25). Notably, several structurally distinct compounds have been identified to inhibit flavivirus replication by intensively targeting the TMD of NS4B (26–32). It is thus conceivable that inhibitors targeting TMD of NS4B would perturb its function, leading to the suppression of viral RNA replication. In this study, the replacement of Q130 of NS4B with a basic amino acid conferred the resistance effect without suppressing JEV replication, suggesting that position 130 could tolerate a basic amino acid and that the basic amino acid might be involved in the interplay of NS4B with host proteins rather than viral proteins.

Moreover, the efficacy and toxicity of manidipine were monitored *in vivo*, with manidipine demonstrating effective antiviral activity with favorable biocompatibility. However, the dose used in this study was higher than the dose typically used clinically, representing one of the scenarios most commonly encountered in drug repurposing (33, 34). As manidipine was approved for use for the long-term treatment of hypertension (35, 36), pulse-dose treatment with manidipine over the shorter period of time required for the treatment of virus infection might be relatively safe. Moreover, use of a combination of manidipine with other Ca^2+^ inhibitors might improve its therapeutic efficacy, reduce its toxicity, and reduce the risk of resistance development (37–39).

Besides the three VGCC inhibitors, two hit drugs, pimecrolimus and nelfinavir mesylate, showed equivalent inhibitory activities on the replication of JEV, ZIKV, WNV, and DENV-2. Although there has been no report on the use of pimecrolimus for the treatment of infectious diseases, we showed that it had a robust effect against JEV with an SI of >32. The maximum plasma concentration (*C*_max_) of nelfinavir mesylate achieved with an adult dose was 3 to 4 μg/ml (40), which was comparable to the IC_50_ reported here. Notably, nelfinavir mesylate was confirmed to inhibit herpes simplex virus 1 (HSV-1) and the replication of several other herpesviruses by interfering directly or indirectly with the later steps of virus formation, such as glycoprotein maturation or virion release, other than functioning in herpesviruses protease (41, 42). Whether nelfinavir mesylate inhibits flavivirus by interference with the virus protease or by other off-target effects is unknown. Understanding of the mechanism of the antiflavivirus effects of these drugs might uncover novel targets of the drugs, providing further insight into the pathogenesis of flaviviruses.

Above all, the findings reported here provide novel insights into the molecular mechanisms underlying flavivirus infection and offer new and promising therapeutic possibilities for combating infections caused by flaviviruses.

## MATERIALS AND METHODS

### Cells and viruses.

BHK-21, SH-SY5Y (human neuroblastoma), Vero, and Huh-7 cells were cultured in Dulbecco modified Eagle medium (HyClone, Logan, UT, USA) supplemented with 10% fetal bovine serum (Gibco, Grand Island, NY, USA). JEV strain AT31, the WNV replicon, and the DENV-2 replicon expressing Renilla luciferase (Rluc) were kindly provided by Bo Zhang, Wuhan Institute of Virology, Chinese Academy of Sciences (CAS), China. JEV replicon recombinant viral particles (RVPs) were generated as previously described (4, 5). ZIKV strain H/PF/2013, kindly provided by the European Virus Archive Goes Global, was propagated and titrated in Vero cells.

### Optimization of HTS assay conditions.

The cell density and RVP dose were optimized for the HTS assay. Vero cells at different densities (2,500 to 12,500 cells per well) were infected with from 1.25 to 20 μl RVPs (1 to 16 copies per well). The appropriate cell density as well as the RVP dose was selected by comparing the S/B ratio, CV, and *Z*′ values under different conditions as previously described (43). Methyl-β-cyclodextrin and dimethyl sulfoxide (DMSO) were used as positive and negative controls, respectively.

### HTS assay of an FDA-approved compound library.

A library of 1,018 FDA-approved drugs was purchased from Selleck Chemicals (Houston, TX, USA). The compounds were stored as 10 mM stock solutions in DMSO at 4°C until use. The first round of the HTS assay was carried out as shown in Fig. 1A. The criteria used to identify the primary candidates were no apparent cytotoxicity and an average level of inhibition of >90% in duplicate wells. The criteria of dose-dependent inhibition and cell viability of >80% were applied for the reconfirmation screen. Furthermore, the CC_50_ of each compound was calculated, and those compounds displaying SIs over 10 were considered hits in this study.

### Identification of antiviral effects of five hit drugs.

The antiviral effects of the drugs were evaluated by quantitative reverse transcription-PCR (qRT-PCR), immunofluorescence assay (IFA), and plaque assay as previously reported (44–47). The experimental timeline is depicted in Fig. 2A.

To ensure the effectiveness of the hit drugs in flavivirus replication, BHK-21 cells transfected with the JEV, WNV, or DENV-2 replicon were incubated with each drug at the concentrations indicated above, and the luciferase activities were determined 24 h, 48 h, or 72 h later, respectively.

### Time-of-addition experiment.

To evaluate which stage of the JEV life cycle was inhibited by each hit, a time-of-addition experiment was performed as previously described (43). Vero cells were infected with 20 μl RVPs for 1 h (0 to 1 h). The test compounds were incubated with the cells for 1 h before infection (−1 to 0 h), during infection (0 to 1 h), and for 23 h postinfection (1 to 24 h) (Fig. 3A). To exclude a possible direct inactivating effect of the drugs, RVPs were incubated with each drug at 37°C for 1 h, and the mixtures were diluted 25-fold to infect Vero cells. Twenty-four hours later, the luciferase activities were determined as described above (Fig. 3A).

### Manidipine-resistant virus.

Manidipine-resistant virus was generated by passaging of JEV on Vero cells in the presence of manidipine. Passages 1 to 10 used 5 μM manidipine, and passages 11 to 20 used 10 μM manidipine. As a control, WT virus was passaged in the presence of 2% DMSO in parallel. Passaging was terminated at passage 20, when no further improvement in resistance was detected. Two manidipine-resistant virus isolates were plaque purified and amplified in the presence of manidipine. Viral RNA was extracted, amplified, and purified for sequencing. An infectious cDNA clone of JEV, strain AT31 (pMWJEAT), kindly provided by T. Wakita, Tokyo Metropolitan Institute for Neuroscience, was used to recover WT and mutant viruses as described previously (4). Virus titers and manidipine sensitivities were determined by plaque assay in Vero cells.

### Manidipine administration to JEV-infected mice.

Adult female BALB/c mice (age, 4 weeks) were kept in the Laboratory Animal Center of Wuhan Institute of Virology, CAS (Wuhan, China). The mice were randomly divided into four groups (30 mice per group): a JEV-infected and vehicle (2% Tween 80 plus 5% DMSO in phosphate-buffered saline [PBS])-treated group, a manidipine-treated group, a JEV-infected and manidipine-treated group, and a vehicle-treated group. For infection, mice were infected intraperitoneally with 5 × 10^6^ PFU of JEV strain AT31. For the manidipine and vehicle treatments, mice were injected intraperitoneally with 25 mg/kg of body weight manidipine or PBS with 2% Tween 80 and 5% DMSO, respectively. Treatments were administered twice a day for the first 2 days and then consecutively administered once a day for up to 21 days. Five mice from each group were sacrificed on days 1, 3, and 5 postinfection. Serum, spleen tissue, and brain tissue samples were collected for viral titer determination and histopathology investigation. Fifteen mice were monitored daily for morbidity and mortality. The mice that showed neurological signs of disease were euthanized according to the Regulations for the Administration of Affairs Concerning Experimental Animals in China. The protocols were reviewed and approved by the Laboratory Animal Care and Use Committee at the Wuhan Institute of Virology, CAS (Wuhan, China).

## References

[1] Mackenzie JS, Gubler DJ, Petersen LR 2004 Emerging flaviviruses: the spread and resurgence of Japanese encephalitis, West Nile and dengue viruses. Nat Med 10:S98–S109. doi:10.1038/nm1144. 15577938

[2] Ishikawa T, Yamanaka A, Konishi E 2014 A review of successful flavivirus vaccines and the problems with those flaviviruses for which vaccines are not yet available. Vaccine 32:1326–1337. doi:10.1016/j.vaccine.2014.01.040. 24486372

[3] Li K, Phoo WW, Luo D 2014 Functional interplay among the flavivirus NS3 protease, helicase, and cofactors. Virol Sin 29:74–85. doi:10.1007/s12250-014-3438-6. 24691778 PMC8206430

[4] Liu H, Liu Y, Wang S, Zhang Y, Zu X, Zhou Z, Zhang B, Xiao G 2015 Structure-based mutational analysis of several sites in the E protein: implications for understanding the entry mechanism of Japanese encephalitis virus. J Virol 89:5668–5686. doi:10.1128/JVI.00293-15. 25762738 PMC4442514

[5] Wang S, Liu H, Zu X, Liu Y, Chen L, Zhu X, Zhang L, Zhou Z, Xiao G, Wang W 2016 The ubiquitin-proteasome system is essential for the productive entry of Japanese encephalitis virus. Virology 498:116–127. doi:10.1016/j.virol.2016.08.013. 27567260

[6] Gagliano T, Gentilin E, Benfini K, Di Pasquale C, Tassinari M, Falletta S, Feo C, Tagliati F, Uberti ED, Zatelli MC 2014 Mitotane enhances doxorubicin cytotoxic activity by inhibiting P-gp in human adrenocortical carcinoma cells. Endocrine 47:943–951. doi:10.1007/s12020-014-0374-z. 25096913

[7] de Wilde AH, Jochmans D, Posthuma CC, Zevenhoven-Dobbe JC, van Nieuwkoop S, Bestebroer TM, van den Hoogen BG, Neyts J, Snijder EJ 2014 Screening of an FDA-approved compound library identifies four small-molecule inhibitors of Middle East respiratory syndrome coronavirus replication in cell culture. Antimicrob Agents Chemother 58:4875–4884. doi:10.1128/AAC.03011-14. 24841269 PMC4136071

[8] Palit P, Ali N 2008 Oral therapy with amlodipine and lacidipine, 1,4-dihydropyridine derivatives showing activity against experimental visceral leishmaniasis. Antimicrob Agents Chemother 52:374–377. doi:10.1128/AAC.00522-07. 17954702 PMC2223878

[9] Bagan J, Compilato D, Paderni C, Campisi G, Panzarella V, Picciotti M, Lorenzini G, Di Fede O 2012 Topical therapies for oral lichen planus management and their efficacy: a narrative review. Curr Pharm Des 18:5470–5480. doi:10.2174/138161212803307617. 22632394

[10] Patick AK, Boritzki TJ, Bloom LA 1997 Activities of the human immunodeficiency virus type 1 (HIV-1) protease inhibitor nelfinavir mesylate in combination with reverse transcriptase and protease inhibitors against acute HIV-1 infection in vitro. Antimicrob Agents Chemother 41:2159–2164. 9333041 10.1128/aac.41.10.2159 PMC164086

[11] Mercorelli B, Luganini A, Nannetti G, Tabarrini O, Palu G, Gribaudo G, Loregian A 2016 Drug repurposing approach identifies inhibitors of the prototypic viral transcription factor IE2 that block human cytomegalovirus replication. Cell Chem Biol 23:340–351. doi:10.1016/j.chembiol.2015.12.012. 26877023

[12] Artunduaga Bonilla JJ, Paredes Guerrero DJ, Sanchez Suarez CI, Ortiz Lopez CC, Torres Saez RG 2015 In vitro antifungal activity of silver nanoparticles against fluconazole-resistant Candida species. World J Microbiol Biotechnol 31:1801–1809. doi:10.1007/s11274-015-1933-z. 26335058

[13] Osorio Y, Travi BL, Renslo AR, Peniche AG, Melby PC 2011 Identification of small molecule lead compounds for visceral leishmaniasis using a novel ex vivo splenic explant model system. PLoS Negl Trop Dis 5:e962. doi:10.1371/journal.pntd.0000962. 21358812 PMC3039689

[14] Puig-Basagoiti F, Deas TS, Ren P, Tilgner M, Ferguson DM, Shi PY 2005 High-throughput assays using a luciferase-expressing replicon, virus-like particles, and full-length virus for West Nile virus drug discovery. Antimicrob Agents Chemother 49:4980–4988. doi:10.1128/AAC.49.12.4980-4988.2005. 16304161 PMC1315944

[15] Sakurai Y, Kolokoltsov AA, Chen CC, Tidwell MW, Bauta WE, Klugbauer N, Grimm C, Wahl-Schott C, Biel M, Davey RA 2015 Ebola virus. Two-pore channels control Ebola virus host cell entry and are drug targets for disease treatment. Science 347:995–998. doi:10.1126/science.1258758. 25722412 PMC4550587

[16] Ding W, Albrecht B, Kelley RE, Muthusamy N, Kim SJ, Altschuld RA, Lairmore MD 2002 Human T-cell lymphotropic virus type 1 p12(I) expression increases cytoplasmic calcium to enhance the activation of nuclear factor of activated T cells. J Virol 76:10374–10382. doi:10.1128/JVI.76.20.10374-10382.2002. 12239314 PMC136546

[17] Barrows NJ, Campos RK, Powell ST, Prasanth KR, Schott-Lerner G, Soto-Acosta R, Galarza-Munoz G, McGrath EL, Urrabaz-Garza R, Gao J, Wu P, Menon R, Saade G, Fernandez-Salas I, Rossi SL, Vasilakis N, Routh A, Bradrick SS, Garcia-Blanco MA 2016 A screen of FDA-approved drugs for inhibitors of Zika virus infection. Cell Host Microbe 20:259–270. doi:10.1016/j.chom.2016.07.004. 27476412 PMC4993926

[18] Scherbik SV, Brinton MA 2010 Virus-induced Ca^2+^ influx extends survival of West Nile virus-infected cells. J Virol 84:8721–8731. doi:10.1128/JVI.00144-10. 20538858 PMC2918993

[19] Chami M, Oules B, Paterlini-Brechot P 2006 Cytobiological consequences of calcium-signaling alterations induced by human viral proteins. Biochim Biophys Acta 1763:1344–1362. doi:10.1016/j.bbamcr.2006.09.025. 17059849

[20] Nagata N, Iwata-Yoshikawa N, Hayasaka D, Sato Y, Kojima A, Kariwa H, Takashima I, Takasaki T, Kurane I, Sata T, Hasegawa H 2015 The pathogenesis of 3 neurotropic flaviviruses in a mouse model depends on the route of neuroinvasion after viremia. J Neuropathol Exp Neurol 74:250–260. doi:10.1097/NEN.0000000000000166. 25668565

[21] Li F, Wang Y, Yu L, Cao S, Wang K, Yuan J, Wang C, Wang K, Cui M, Fu ZF 2015 Viral infection of the central nervous system and neuroinflammation precede blood-brain barrier disruption during Japanese encephalitis virus infection. J Virol 89:5602–5614. doi:10.1128/JVI.00143-15. 25762733 PMC4442524

[22] Lavanya M, Cuevas CD, Thomas M, Cherry S, Ross SR 2013 siRNA screen for genes that affect Junin virus entry uncovers voltage-gated calcium channels as a therapeutic target. Sci Transl Med 5:204ra131. doi:10.1126/scitranslmed.3006827. PMC4104171 24068738

[23] Han Z, Madara JJ, Herbert A, Prugar LI, Ruthel G, Lu J, Liu Y, Liu W, Liu X, Wrobel JE, Reitz AB, Dye JM, Harty RN, Freedman BD 2015 Calcium regulation of hemorrhagic fever virus budding: mechanistic implications for host-oriented therapeutic intervention. PLoS Pathog 11:e1005220. doi:10.1371/journal.ppat.1005220. 26513362 PMC4634230

[24] Selisko B, Wang C, Harris E, Canard B 2014 Regulation of flavivirus RNA synthesis and replication. Curr Opin Virol 9:74–83. doi:10.1016/j.coviro.2014.09.011. 25462437 PMC4295515

[25] Munoz-Jordan JL, Laurent-Rolle M, Ashour J, Martinez-Sobrido L, Ashok M, Lipkin WI, Garcia-Sastre A 2005 Inhibition of alpha/beta interferon signaling by the NS4B protein of flaviviruses. J Virol 79:8004–8013. doi:10.1128/JVI.79.13.8004-8013.2005. 15956546 PMC1143737

[26] Guo F, Wu S, Julander J, Ma J, Zhang X, Kulp J, Cuconati A, Block TM, Du Y, Guo J-T, Chang J 2016 A novel benzodiazepine compound inhibits yellow fever virus infection by specifically targeting NS4B protein. J Virol 90:10774–10788. doi:10.1128/JVI.01253-16. PMC5110185 27654301

[27] Patkar CG, Larsen M, Owston M, Smith JL, Kuhn RJ 2009 Identification of inhibitors of yellow fever virus replication using a replicon-based high-throughput assay. Antimicrob Agents Chemother 53:4103–4114. doi:10.1128/AAC.00074-09. 19651907 PMC2764201

[28] Wang QY, Dong H, Zou B, Karuna R, Wan KF, Zou J, Susila A, Yip A, Shan C, Yeo KL, Xu H, Ding M, Chan WL, Gu F, Seah PG, Liu W, Lakshminarayana SB, Kang C, Lescar J, Blasco F, Smith PW, Shi PY 2015 Discovery of dengue virus NS4B inhibitors. J Virol 89:8233–8244. doi:10.1128/JVI.00855-15. 26018165 PMC4524232

[29] de Wispelaere M, LaCroix AJ, Yang PL 2013 The small molecules AZD0530 and dasatinib inhibit dengue virus RNA replication via Fyn kinase. J Virol 87:7367–7381. doi:10.1128/JVI.00632-13. 23616652 PMC3700292

[30] van Cleef KW, Overheul GJ, Thomassen MC, Kaptein SJ, Davidson AD, Jacobs M, Neyts J, van Kuppeveld FJ, van Rij RP 2013 Identification of a new dengue virus inhibitor that targets the viral NS4B protein and restricts genomic RNA replication. Antiviral Res 99:165–171. doi:10.1016/j.antiviral.2013.05.011. 23735301

[31] Xie X, Wang QY, Xu HY, Qing M, Kramer L, Yuan Z, Shi PY 2011 Inhibition of dengue virus by targeting viral NS4B protein. J Virol 85:11183–11195. doi:10.1128/JVI.05468-11. 21865382 PMC3194949

[32] van Cleef KW, Overheul GJ, Thomassen MC, Marjakangas JM, van Rij RP 2016 Escape mutations in NS4B render dengue virus insensitive to the antiviral activity of the paracetamol metabolite AM404. Antimicrob Agents Chemother 60:2554–2557. doi:10.1128/AAC.02462-15. 26856827 PMC4808173

[33] Cheer SM, McClellan K 2001 Manidipine: a review of its use in hypertension. Drugs 61:1777–1799. doi:10.2165/00003495-200161120-00010. 11693466

[34] McKeage K, Scott LJ 2004 Manidipine: a review of its use in the management of hypertension. Drugs 64:1923–1940. doi:10.2165/00003495-200464170-00011. 15329044

[35] Roca-Cusachs A, Triposkiadis F 2005 Antihypertensive effect of manidipine. Drugs 65(Suppl 2):S11–S19. 10.2165/00003495-200565002-00003 16398058

[36] Mizuno K, Haga H, Takahashi M, Watanabe Y, Fukuchi S 1992 Clinical evaluation of the efficacy and safety of manidipine in hypertensive patients with renal disorders. Blood Pressure Suppl 3:119–123. 1343279

[37] Otero ML 2007 Manidipine-delapril combination in the management of hypertension. Vasc Health Risk Manag 3:255–263. 17703633 PMC2293964

[38] Johansen LM, DeWald LE, Shoemaker CJ, Hoffstrom BG, Lear-Rooney CM, Stossel A, Nelson E, Delos SE, Simmons JA, Grenier JM, Pierce LT, Pajouhesh H, Lehar J, Hensley LE, Glass PJ, White JM, Olinger GG 2015 A screen of approved drugs and molecular probes identifies therapeutics with anti-Ebola virus activity. Sci Transl Med 7:290ra289. doi:10.1126/scitranslmed.aaa5597. 26041706

[39] Lehar J, Krueger AS, Avery W, Heilbut AM, Johansen LM, Price ER, Rickles RJ, Short GF III, Staunton JE, Jin X, Lee MS, Zimmermann GR, Borisy AA 2009 Synergistic drug combinations tend to improve therapeutically relevant selectivity. Nat Biotechnol 27:659–666. doi:10.1038/nbt.1549. 19581876 PMC2708317

[40] Jackson KA, Rosenbaum SE, Kerr BM, Pithavala YK, Yuen G, Dudley MN 2000 A population pharmacokinetic analysis of nelfinavir mesylate in human immunodeficiency virus-infected patients enrolled in a phase III clinical trial. Antimicrob Agents Chemother 44:1832–1837. doi:10.1128/AAC.44.7.1832-1837.2000. 10858338 PMC89969

[41] Gantt S, Carlsson J, Ikoma M, Gachelet E, Gray M, Geballe AP, Corey L, Casper C, Lagunoff M, Vieira J 2011 The HIV protease inhibitor nelfinavir inhibits Kaposi's sarcoma-associated herpesvirus replication in vitro. Antimicrob Agents Chemother 55:2696–2703. doi:10.1128/AAC.01295-10. 21402841 PMC3101462

[42] Kalu NN, Desai PJ, Shirley CM, Gibson W, Dennis PA, Ambinder RF 2014 Nelfinavir inhibits maturation and export of herpes simplex virus 1. J Virol 88:5455–5461. doi:10.1128/JVI.03790-13. 24574416 PMC4019105

[43] Fang J, Sun L, Peng G, Xu J, Zhou R, Cao S, Chen H, Song Y 2013 Identification of three antiviral inhibitors against Japanese encephalitis virus from library of pharmacologically active compounds 1280. PLoS One 8:e78425. doi:10.1371/journal.pone.0078425. 24348901 PMC3857149

[44] Jin R, Zhu W, Cao S, Chen R, Jin H, Liu Y, Wang S, Wang W, Xiao G 2013 Japanese encephalitis virus activates autophagy as a viral immune evasion strategy. PLoS One 8:e52909. doi:10.1371/journal.pone.0052909. 23320079 PMC3540057

[45] Zu X, Liu Y, Wang S, Jin R, Zhou Z, Liu H, Gong R, Xiao G, Wang W 2014 Peptide inhibitor of Japanese encephalitis virus infection targeting envelope protein domain III. Antiviral Res 104:7–14. doi:10.1016/j.antiviral.2014.01.011. 24468276

[46] Chen L, Liu Y, Wang S, Sun J, Wang P, Xin Q, Zhang L, Xiao G, Wang W 2017 Antiviral activity of peptide inhibitors derived from the protein E stem against Japanese encephalitis and Zika viruses. Antiviral Res 141:140–149. doi:10.1016/j.antiviral.2017.02.009. 28232248

[47] Xin QL, Deng CL, Chen X, Wang J, Wang SB, Wang W, Deng F, Zhang B, Xiao G, Zhang LK 2017 Quantitative proteomic analysis of mosquito C6/36 cells reveals host proteins involved in Zika virus infection. J Virol 91:e00554-17. doi:10.1128/JVI.00554-17. 28404849 PMC5446628

[48] Miller S, Sparacio S, Bartenschlager R 2006 Subcellular localization and membrane topology of the dengue virus type 2 non-structural protein 4B. J Biol Chem 281:8854–8863. doi:10.1074/jbc.M512697200. 16436383

